# Comparative transcriptome analysis of persimmon somatic mutants (*Diospyros kaki*) identifies regulatory networks for fruit maturation and size

**DOI:** 10.3389/fpls.2024.1448851

**Published:** 2024-08-02

**Authors:** Seunghyun Ban, Hye-young Suh, Su Hyeon Lee, Si-Hong Kim, Sewon Oh, Je Hyeong Jung

**Affiliations:** ^1^ Department of Horticulture, College of Agriculture and Life Science, Kyungpook National University, Daegu, Republic of Korea; ^2^ World Horti Center, Kyungpook National University, Sangju, Republic of Korea; ^3^ Sangju Persimmon Research Institute, Gyeongsangbuk-do Agricultural Research and Extension Services, Sangju, Republic of Korea; ^4^ Smart Farm Research Center, Korea Institute of Science and Technology (KIST), Gangneung, Republic of Korea; ^5^ Fruit Research Division, National Institute of Horticultural and Herbal Science, Rural Development Administration, Wanju, Republic of Korea

**Keywords:** bud sports, persimmon, comparative transcriptome analysis, fruit maturation, fruit size

## Abstract

Bud sports in fruit crops often result in new cultivars with unique traits, such as distinct fruit size and color, compared to their parent plants. This study investigates the phenotypic differences and gene expression patterns in Tonewase and Ohtanenashi persimmon bud sports compared to those in their parent, Hiratanenashi, based on RNA-seq data. Tonewase is characterized by early maturation, whereas Ohtanenashi is noted for its larger fruit size. Despite the importance of these traits in determining fruit quality, their molecular bases in persimmons have been understudied. We compared transcriptome-level differences during fruit development between the bud sport samples and their original cultivar. Comprehensive transcriptome analyses identified 15,814 differentially expressed genes and 26 modules via weighted gene co-expression network analysis. Certain modules exhibited unique expression patterns specific to the different cultivars during fruit development, likely contributing to the phenotypic differences observed. Specifically, M11, M16, M22, and M23 were uniquely expressed in Tonewase, whereas M13 and M24 showed distinct patterns in Ohtanenashi. By focusing on genes with distinct expression profiles, we aimed to uncover the genetic basis of cultivar-specific traits. Our findings suggest that changes in the expression of genes associated with ethylene and cell wall pathways may drive Tonewase’s earlier maturation, whereas genes related to the cell cycle within the M24 module appear crucial for Ohtanenashi’s larger fruit size. Additionally, ethylene and transcription factor genes within this module may contribute to the increased fruit size observed. This study elucidates the differences in transcriptomic changes during fruit development between the two bud sport samples and their original cultivar, enhancing our understanding of the genetic determinants influencing fruit size and maturation.

## 1 Introduction

The persimmon (*Diospyros kaki* Thunb.), a member of the Ebenaceae family, is a significant fruit tree originating from Eastern Asia. According to the Food and Agriculture Organization (2023), global production reached 4,436,475 tons in 2022, covering 1,044,386 hectare of land (FAO, 2023). The top five producers—China, Republic of Korea, Japan, Azerbaijan, and Brazil—dominate the market, with China alone contributing 76% of total production. Collectively, these five countries account for 94.5% of global persimmon production. Persimmons are broadly categorized into non-astringent and astringent types. Horticulturally, they are further classified into four types on the basis of astringency loss traits related to seed presence and the influence of pollination on flesh color: pollination constant non-astringent, pollination variant non-astringent, pollination constant astringent, and pollination variant astringent (PVA) (Sato and Yamada, 2016). More than a thousand persimmon varieties have been identified, with notable cultivars including Fuyu, Jiro, and Hiratanenashi (Tao and Luo, 2022). Although research on astringency loss traits has traditionally been a central focus in persimmon studies, with numerous experiments aimed at elucidation (Mo et al., 2016; Chen et al., 2017; Guan et al., 2017; Nishiyama et al., 2018; Wang et al., 2022), other important traits such as fruit size and maturation date have received less attention.

Bud sports in fruit crops refer to mutations where parts of a plant, such as flowers or fruits, exhibit phenotypes distinct from the parent plant (Foster and Aranzana, 2018). Numerous bud sports have been reported, showing variations in horticulturally important traits such as fruit size (Nashima et al., 2014; Huang et al., 2023), color (Kobayashi et al., 2004; Alos et al., 2008; El-Sharkawy et al., 2015), maturation date (Wang et al., 2009; Ban et al., 2022), fruit-bearing type (Han et al., 2017), tree growth habit (Otto et al., 2013; Wolters et al., 2013; Okada et al., 2016), seedlessness (Nwafor et al., 2014; Royo et al., 2018), and fruit shape (Lopez-Girona et al., 2017; Tan et al., 2019). These bud sports can be propagated using methods such as grafting, thereby enabling the development of new varieties. The advantages of bud sports in fruit crops include overcoming challenges in traditional breeding, such as a lengthy juvenile period and self-incompatibility, contributing to their high commercial value. Additionally, bud sports hold substantial scientific value because molecular differences identified through comparison with the original variety can help uncover the underlying molecular mechanisms of their distinct traits. For instance, seedless traits are highly desirable for the consumption of fresh fruit in grapes (Varoquaux et al., 2000). A comparative analysis of transcriptome changes during fruit development between a seedless bud sports grape and the original seeded wine cultivar revealed variations in the expression of genes associated with the pollen and ovule developmental pathways (Nwafor et al., 2014). This study further suggested that the identified genes could be responsible for the seedless phenotype, opening up the possibility to achieve the seedless trait through genetic mechanism analysis. Previously, this trait was induced artificially during the flowering period using growth regulators like gibberellic acid (Naito et al., 1974; Lu et al., 1997). Moreover, bud sports serve as valuable research materials for investigating the influence of diverse gene networks on maturation and fruit size throughout the various stages of fruit development. For example, a comparative transcriptome profiling study conducted on the late-ripening sweet orange mutant of ‘Jincheng’ (*Citrus sinensis* L. Osbeck) and its wild type identified 13,412 differentially expressed genes (DEGs) (Zhang et al., 2014). These DEGs predominantly clustered into five pathways: metabolic pathways, plant-pathogen interaction, spliceosome, biosynthesis of plant hormones, and biosynthesis of phenylpropanoids, underscoring their significant impact on fruit development. Another example is a transcriptome analysis conducted on a bud sport displaying a large berry phenotype and its wild type, which identified DEGs associated with cell wall modification, stress response, and the cell killing pathway, suggesting that these genes might be responsible for the observed phenotypic variation (Zhang et al., 2014).

Hiratanenashi, a nonaploid (*2n* = 135 = 9x) persimmon cultivar, is a PVA and seedless variety, making it the leading cultivar in Japan (Yakushiji et al., 2016). It is recognized for its astringent fruit that loses astringency after harvest. Several bud sports originating from the Hiratanenashi cultivar exhibit diverse phenotypic variation. For instance, Hasshu produces smaller, dwarf-like fruit size with seeds (Yakushiji et al., 2016), whereas Totsutanenashi results in smaller fruit with fewer and smaller cells (Habu et al., 2016). Conversely, Ohtanenashi is characterized by larger fruits (Giordani, 2002). Additionally, some bud sports have different maturation dates; for example, Ishibashiwase and Tonewase exhibit accelerated maturation. Tonewase has become particularly popular, with its cultivation area (2,170.5 ha) nearing that of the original cultivar (2,268.9 ha) as of 2022 (Tao and Luo, 2022).

Although numerous bud sports have been documented in Hiratanenashi, research on the molecular mechanisms driving these phenotypic differences remains limited. Understanding traits like fruit size and maturation is essential as they greatly influence the marketability and cultivation methods of persimmons. To address this gap, we performed a comparative transcriptomic analysis of the original Hiratanenashi cultivar and its bud sports, Tonewase, which matures early, and Ohtanenashi, which produces larger fruits. Our objective was to uncover the genetic basis for these traits by examining transcriptomic changes from early fruit development stages through to maturation across the three cultivars.

## 2 Materials and methods

### 2.1 Plant materials

Tonewase and Ohtanenashi buds, originating from Hiratanenashi bud mutations, exhibit distinct characteristics. Tonewase is known for its early maturation, occurring 10–15 days ahead of the standard maturation period, whereas Ohtanenashi stands out for its large fruit size (Giordani, 2002). We conducted a genetic analysis to validate the parent-sport relationship between these two bud mutations and the original (wild-type) cultivar. Genomic DNA (gDNA) was extracted from the young leaves of 192 samples (**Supplementary Table S1**) using the modified Cetyltrimethylammonium bromide (CTAB) method (Doyle and Doyle, 1987). Three biological replicates for Hiratanenashi, Tonewase, and Ohtanenashi were intentionally included from the trees used for fruit sampling during fruit development.

From 3 weeks after full bloom (WAFB) until the maturation date in 2022, we monitored the fruit development processes in three cultivars: Hiratanenashi, enlarged fruit cultivar Ohtanenashi, and the early maturation cultivar Tonewase. We selected three trees of each cultivar at the Sangju Persimmon Research Institute (Gyeongsangbuk-do, Sangju, Korea) to ensure that the selected trees did not display distinct differences in size. All samples were managed using the same conventional cultivation practices at the Sangju Persimmon Research Institute. For Hiratanenashi and Ohtanenashi, fruit sampling occurred at six different points, whereas Tonewase, which is known for its early maturation, was sampled at five points, excluding the last one among the six sampling points. At each sampling event, 15 fruits were collected and used for phenotyping. Subsequently, these fruits were divided into two biological replicates. These collected fruits were rapidly frozen using liquid nitrogen and stored at temperatures below −80°C until use in subsequent analyses.

### 2.2 Flow cytometry

About 30 mg of young leaf tissue was finely chopped with a razor blade in a glass Petri dish containing 1 mL of Otto I solution (0.1 M citric acid, 0.5% v/v Tween 20) and stained for 60 s. The resulting crude homogenate was then filtered into the sample tube through a 50-µm nylon mesh, followed by staining with 2 mL of a 4,6-diamino-2-phenylindole dihydrochloride solution for 1 min. Subsequently, the relative fluorescence intensity of the stained nuclei was assessed using a flow cytometer (CyFlow Ploidy Analyser) in the UV channel. The ploidy levels of two bud sport persimmon cultivars were assessed by comparing the fluorescence values between the peak positions of the samples with those of its parent. *Diospyros lotus* (2n = 2x) and Sangjudungsi (2n = 6x) were employed as reference standards (**Supplementary Figure S1**).

### 2.3 Phenotyping

For this study, we selected 15 samples from each of the three cultivars at every sampling time point to measure their weight and analyze phenotypic characteristics. To enhance the robustness and reliability of our measurements, we obtained RGB values from three independent replicates for each fruit. This approach utilized the color-summarizer image tool provided by the Genome Science Center (http://mkweb.bcgsc.ca/color-summarizer/), which allowed us to extract and quantify the red, green, and blue values of the RGB color space from the images captured. By averaging these replicates, we derived a more accurate representative value for each fruit’s color characteristic, reflecting a comprehensive assessment of the RGB color values across the fruit surface.

### 2.4 DNA library construction for genotyping by sequencing

Freshly extracted gDNA from 192 samples was evaluated for concentration and quality using a Thermo Scientific Nanodrop 2000 spectrophotometer (Fisher Scientific, Waltham, MA, USA) and 1.5% agarose gel. To prepare genotyping-by-sequencing (GBS) libraries, gDNA from 192 samples was digested with the restriction enzyme *ApeK* I (G/CWGC). Subsequently, the processed samples were divided into two sets of 96 samples each, and ligation was performed using individual barcode adapters for each set (Elshire et al., 2011). Each plate was then sequenced with 151–base pair reads using TruSeq version 3.0, paired-end sequencing on the Illumina HiSeq X Ten platform (Illumina, Inc., San Diego, CA, USA).

### 2.5 Sequencing and SNP genotyping

Reads containing only the common adapter were removed following sequencing using the Cutadapt v1.8.3 software (Martin, 2011). Subsequently, the raw reads were de-multiplexed using a Python script, which excluded reads with ambiguous bases in the barcodes and separated the Illumina FASTQ file into 192 individual files based on barcode sequences (Eun et al., 2016). After de-multiplexing, the reads were trimmed using the Solexa QA package v.1.13 (Eun et al., 2016), discarding poor-quality reads (Phred score less than 20) and those shorter than 25 bases.

The Burrows–Wheeler Aligner program (0.7.17-r1188) was applied to ensure high mapping quality for reliable variant calling for alignment to the draft genome sequence of *Diospyros kaki* (*D. kaki*) (Horiuchi et al., 2023) and subsequent Single-nucleotide polymorphism (SNP) calling (Li and Durbin, 2009). Mapped reads were extracted from the resulting BAM file for further analysis using SAM tools v.0.1.16 (Li et al., 2009). SNPs were called at variable positions with a minimal mapping quality (-Q) of 30, and read depths were set between 3 and 218. Significant sites among the called SNP positions were selected using an in-house script, considering biallelic loci (Kim et al., 2014).

A raw SNP matrix was constructed by collecting the raw SNP positions from each sample and comparing them with the reference genome. The blank regions, where no SNPs were identified, were filled from the consensus sequence of the sample. A final SNP matrix was created by filtering out the low-quality SNP loci. SNPs with a minor allele frequency (MAF) greater than 5%, biallelic SNP loci, and missing data in less than 10% of samples were selected.

### 2.6 Phylogenetic tree construction

The phylogenetic tree was constructed using TASSEL5, which employs the neighbor-joining method to compare genetic distances among the 192 samples based on the final selected SNPs obtained through SNP filtering.

### 2.7 Total RNA isolation, library construction, and RNA-seq

A total of 34 samples, comprising two biological replicates for each of the 17 sampling points (six in Hiratanenashi, six in Ohtanenashi, and five in Tonewase), were subjected to total RNA extraction following a previously described protocol (Meisel et al., 2005). The extracted RNA was treated with Ribonuclease (RNase)-free Deoxyribonuclease (DNase) (Qiagen, Valencia, CA, USA) and purified using an RNeasy Mini Kit (Qiagen). Measurements were performed using a NanoDrop 2000, and agarose gel assays were conducted on a 1.0% gel to assess the concentration and quality of RNA. After performing quality control procedures, RNA-seq libraries were constructed according to the manufacturer’s protocol using the TruSeq Stranded mRNA Library Prep Kit. The constructed libraries were multiplexed in equal amounts for subsequent paired-end 101-base sequencing. Sequencing was performed at Macrogen (Seoul, Korea) using the NovaSeq6000 platform (Illumina).

### 2.8 RNA-seq data analysis

The sequencing data underwent demultiplexing, and subsequent reads were trimmed to remove adapter sequences and filter out low-quality reads. This process was conducted using BBDuk within Geneious Prime version 2023.1 (https://www.geneious.com/), with a cutoff threshold set at an average base quality score of 20. After quality control, the filtered reads were mapped to the *D. kaki* reference genome (Horiuchi et al., 2023) using the medium-low sensitivity function of the Geneious RNA mapper. DEGs between two samples were identified by considering an FPKM (fragments per kilobase per million mapped fragments) value of ≥0.5 in at least one of the samples, a mean log2 fold change of ≥0.585, indicating a biologically significant change, and an associated adjusted p-value of ≤0.05, ensuring statistical relevance using DESeq2 method. Comparative analysis was conducted between adjacent developmental stages within each cultivar series to capture expression dynamics across the progression. Additionally, samples within the same developmental stage but across different cultivars were compared to discern varietal expression differences.

### 2.9 Quantitative real-time polymerase chain reaction analyses 

Total RNA identical to that used for RNA sequencing analyses was subjected to DNase I treatment (QIAGEN) to eliminate any contaminated gDNA. First-strand cDNA synthesis was conducted using 1 μg of total RNA and a cDNA synthesis kit (Thermo Fisher Scientific, Waltham, MA, USA). The quantitative real-time PCR (qRT-PCR) reactions were performed using the LightCycler^®^ 480 SYBR Green I Master mix (Roche, Basel, Switzerland). The thermocycling conditions were as follows: a pre-incubation step at 95°C for 5 min, followed by 40 cycles of amplification at 95°C for 10 s, 58°C for 30 s, and 72°C for 1 min. Relative gene expression levels were normalized to Glyceraldehyde-3-phosphate dehydrogenase(GAPDH) (Wang et al., 2020a) expression and calculated using the 2^−ΔΔCt^ method. Primers utilized for the qRT-PCR assays are detailed in **Supplementary Table S2**.

### 2.10 Weighted gene co-expression network analysis

A co-expression network of selected DEGs was constructed using weighted gene co-expression network analysis (WGCNA) (Langfelder and Horvath, 2008). The parameters used for this analysis were as follows: signed topological overlap matrix, power parameter (b) of 9, minimal module size of 20, reassigned threshold of 0.25, and branch-merge cut height of 0.25. The eigengene (representing the first principal component of the module) value was computed for each resulting WGCNA module. This value was used to assess the association between the module and measured phenotype. The correlation network of essential modules was visualized using Cytoscape (v. 3.10.1).

### 2.11 GO term annotation of the persimmon reference genome and gene enrichment analysis

As the *D. kaki* reference genome lacked publicly available Gene Ontology (GO) annotations, we addressed this gap using Blast2GO software (version 6.0) (Conesa et al., 2005) for annotation. Initially, we established a local reference database using RefSeq non-redundant protein files from NCBI (version 2022.12) through the Makeblastdb command. Next, the 39,464 proteins annotated in the *D. kaki* genome underwent a BLAST-P search against the local protein database and the online InterPro collection database. The parameters for this search included an High-scoring segment pair(HSP) cutoff length of 33, reporting 20 hits, and a minimum e-value of 10^−5^. GO terms were assigned to *D. Kaki* proteins based on significant hits in the databases, and associated GO terms were pre-mapped in Blast2GO. Cutoffs encompassed an e-value hit filter of 10^−6^, annotation of 55, GO weight of 5, and HSP-hit coverage of 20.

For GO term enrichment analysis in WGCNA modules, we utilized the “Fisher’s exact test configuration” in Blast2GO, with a cutoff false discovery rate (FDR) P-value of ≤ 0.05. To further infer the putative function of annotated genes, we obtained Kyoto encyclopedia of genes and genomes (KEGG) (Kanehisa and Goto, 2000) terms using eggNOG-MAPPER (Cantalapiedra et al., 2021). Additionally, we conducted enrichment analysis of the identified modules from WGCNA using the Kyoto encyclopedia of genes and genomes(KEGG) enrichment Analyze function in TBtools (version 2.012) (Chen et al., 2020).

## 3 Results

### 3.1 Phenotypes and ploidy analysis of Hiratanenashi and its two mutants, Ohtanenashi and Tonewase

**Figure 1:**
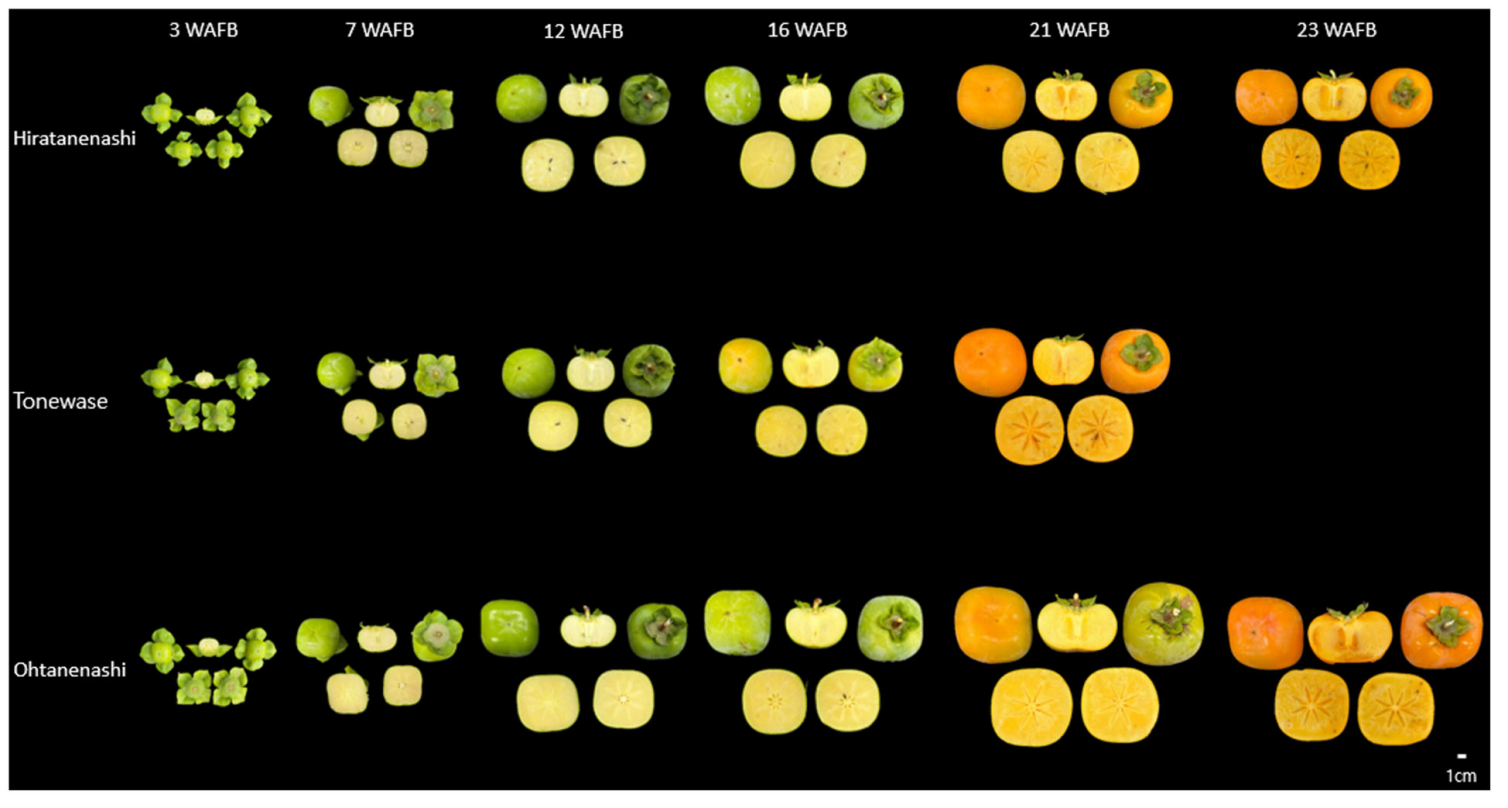
Fruit morphology in Hiratanenashi, Tonewase, and Ohtanenashi during fruit development [23 weeks after full bloom (WAFB)].

**Figure 2:**
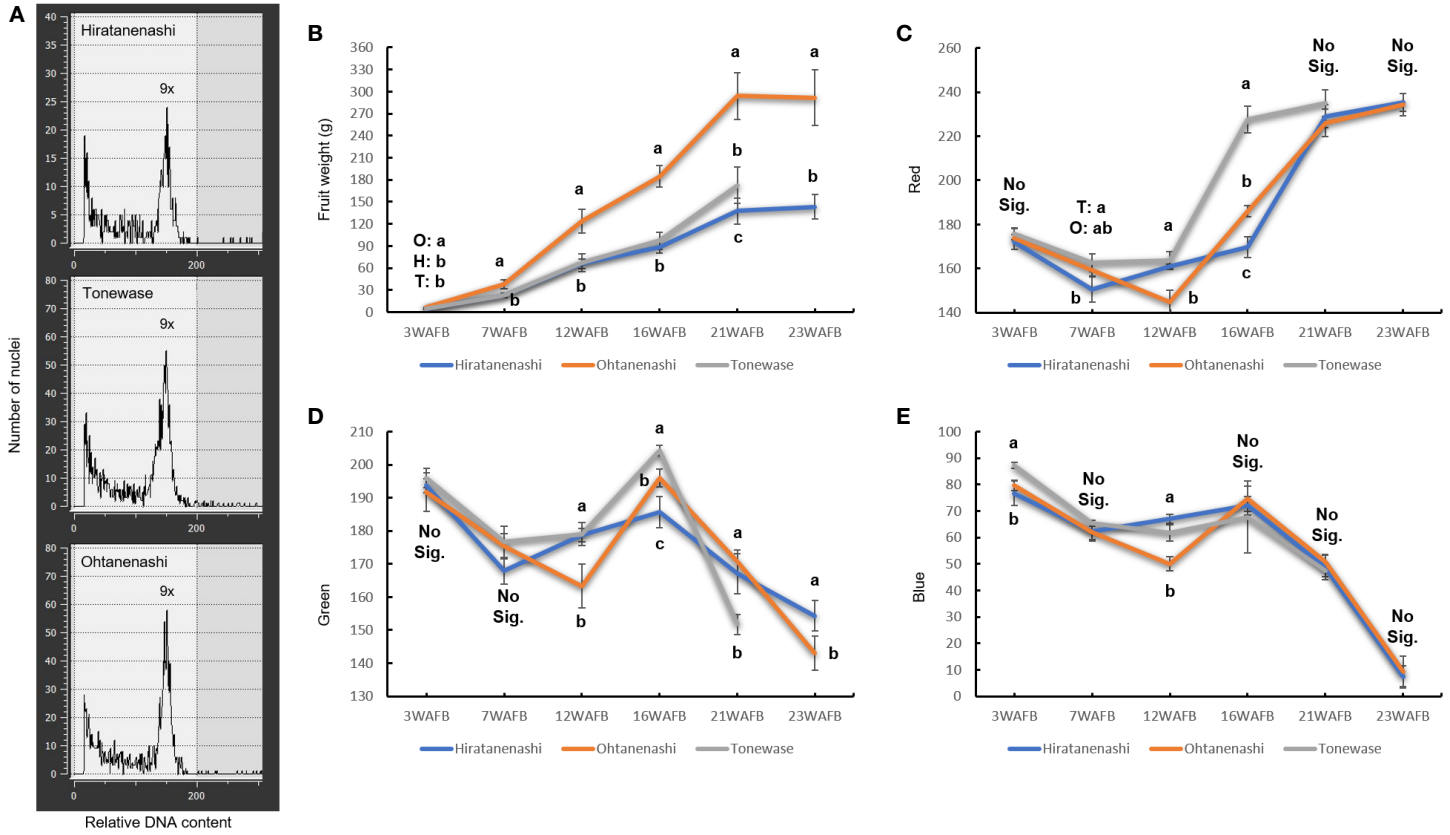
Characteristics of Hiratanenashi, Tonewase, and Ohtanenashi. **(A)** Flow cytometric analyses of nuclear DNA content in young leaves of Hiratanenashi, Tonewase, and Ohtanenashi. Changes in **(B)** fruit weight and **(C–E)** color index. Values in the fruit weight chart represent a mean ± SD of 15 independent biological replicates. Values in the RGB (red, green, and blue) chart represent a mean ± SD of three replicates. Different letters indicate significant differences based on one-way ANOVA and Tukey’s Honestly significant difference (HSD) test (P < 0.05). O represents Ohtanenashi, H represents Hiratanenashi, and T represents Tonewase. Y-axis represents weeks after full bloom (WAFB).

Ohtanenashi consistently exhibited a larger fruit size than Hiratanenashi throughout the early stages of fruit development from 3 WAFB until the harvest stage (**Figures 1**, **2**). Tonewase also showed differences in fruit size compared to Hiratanenashi. No significant differences were observed from the early stages to 16 WAFB; however, a significant difference was observed at 21 WAFB (**Figures 1**, **2**). Ploidy levels were assessed through flow cytometry to determine whether the phenotypic differences between the bud sport and the original cultivar were attributable to variations in ploidy levels (**Figure 2A**, **Supplementary Figure S1**). The fluorescence peaks of Tonewase and Ohtanenashi coincided with those of their parent, Hiratanenashi, indicating no difference in ploidy levels among these three samples.

Fruit maturity was assessed on the basis of color change using RGB color measurements (**Figures 1**, **2**). Tonewase exhibited a red color on the fruit surface as early as 16 WAFB, a feature distinct from that of the other two cultivars (**Figure 2C**). The red color intensity, represented by the R (red) value, rapidly increased from 163.6 at 12 WAFB to 227.6 at 16 WAFB. In contrast, the R values for the other two cultivars remained below 190 during the same period. By 21 WAFB, Tonewase displayed an intensified red color, the rapid disappearance of the green color, and a rich red hue on the fruit surface, indicating that it was ready for harvest (**Figures 2C, D**). In contrast, Hiratanenashi and Ohtanenashi achieved red color levels on the fruit surface similar to what Tonewase had at 16 WAFB by the time that they reached 21 WAFB, and they became harvest-ready at 23 WAFB. In conclusion, Tonewase demonstrated maturation progress approximately 2 weeks faster than the other cultivars.

### 3.2 Genetic evaluation confirming somatic mutant relationship among Hiratanenashi, Ohtanenashi, and Tonewase

**Figure 3:**
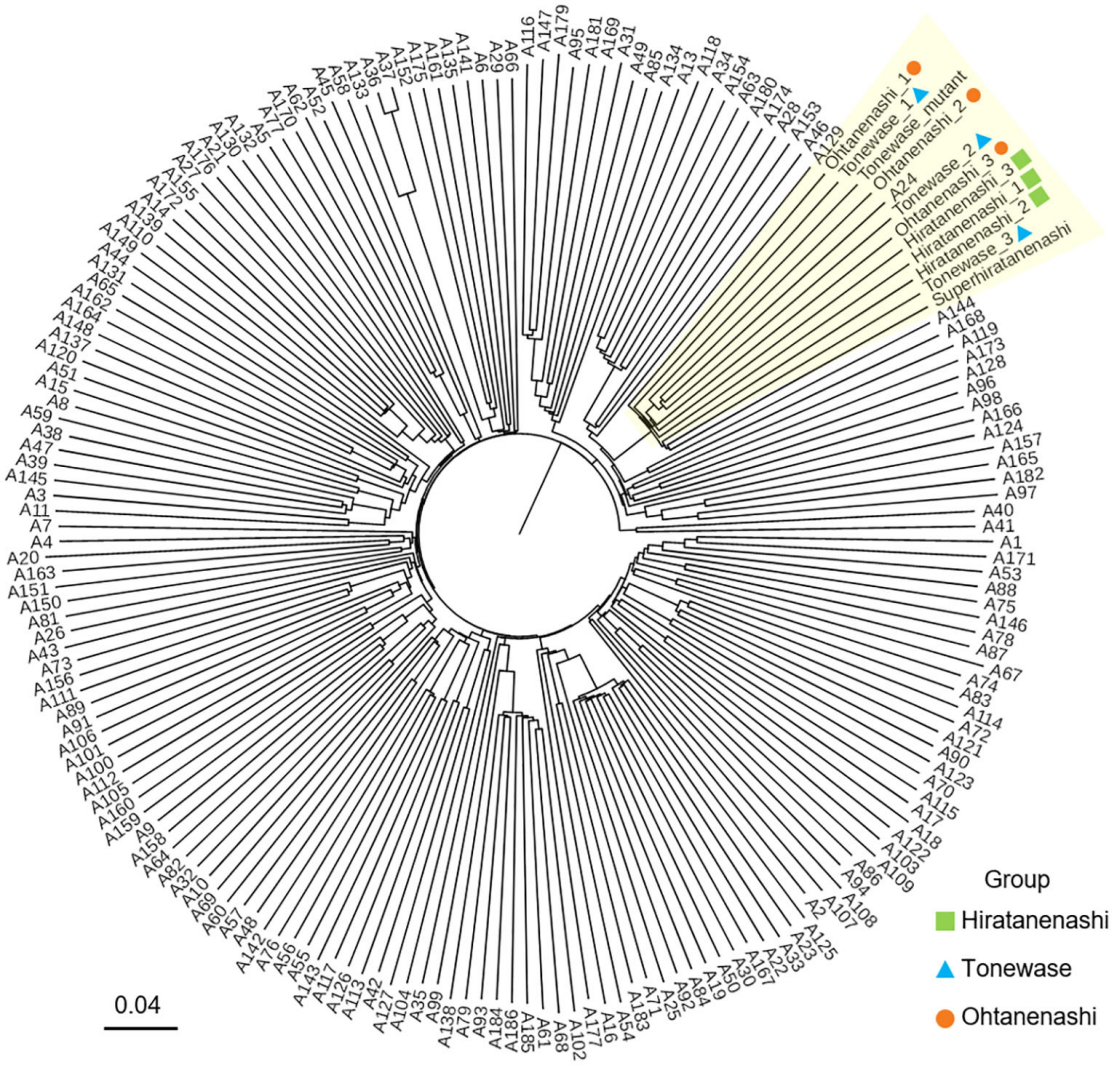
Phylogenetic relationship of 192 persimmon samples. Samples clustered under the same clade with Hiratanenashi, Tonewase, and Ohtanenashi are highlighted in yellow. The phylogenetic tree was constructed with Trait analysis by association, evolution and linkage (TASSEL) using the neighbor-joining method.

We conducted a genetic evaluation of 192 samples from diverse persimmon cultivars using GBS to confirm the somatic mutation origins of Ohtanenashi and Tonewase from Hiratanenashi. While simple sequence repeat (SSR) markers are typically used to test the parent relationship between a bud sport and its original variety, we aimed to perform a more comprehensive analysis at the whole-chromosome level. Our sample set included not only Ohtanenashi and Tonewase but also other bud sports derived from Hiratanenashi, allowing us to compare multiple bud sports simultaneously. The GBS analysis yielded 1,575.7 million raw reads, and 1,350.3 million reads remained after trimming (**Supplementary Table S1**). Of the trimmed reads, 1,240.6 million were successfully mapped to the *D. kaki* reference genome, with an average mapping rate of 91.9%. SNP calling identified SNPs at 2,921,723 loci in 192 samples. Subsequently, SNPs meeting the criteria (MAF > 5% and less than 10% missing data) were selected, resulting in 55,930 SNPs for the phylogenetic tree analysis (**Figure 3**). The analysis revealed that Hiratanenashi, Ohtanenashi, and Tonewase formed distinct clusters. Another mutant of Tonewase (Tonewase_mutant) and a different mutant of Hiratanenashi (SuperHiratanenashi) were grouped in the same clade. This strongly indicates that Hiratanenashi, Ohtanenashi, and Tonewase are indeed in a somatic mutation relationship.

### 3.3 Transcriptomes profiling of Hiratanenashi and its two mutants throughout fruit development

We performed comprehensive RNA-seq analyses across various fruit development stages for three cultivars to uncover the mechanisms driving enhanced fruit size in Ohtanenashi and expedited maturation in Tonewase compared to their parent cultivar, Hiratanenashi. In our study, we generated 34 RNA-seq libraries were generated, covering six different time points for Hiratanenashi and Ohtanenashi (with two replicates each) and five time points for Tonewase (with two replicates each). These libraries collectively yielded 2,521.7 million clean reads (**Supplementary Table S3**), with 2,106.8 million reads (83.5% of the total) successfully aligned and mapped to the reference genome of *D. kaki*.

**Figure 4:**
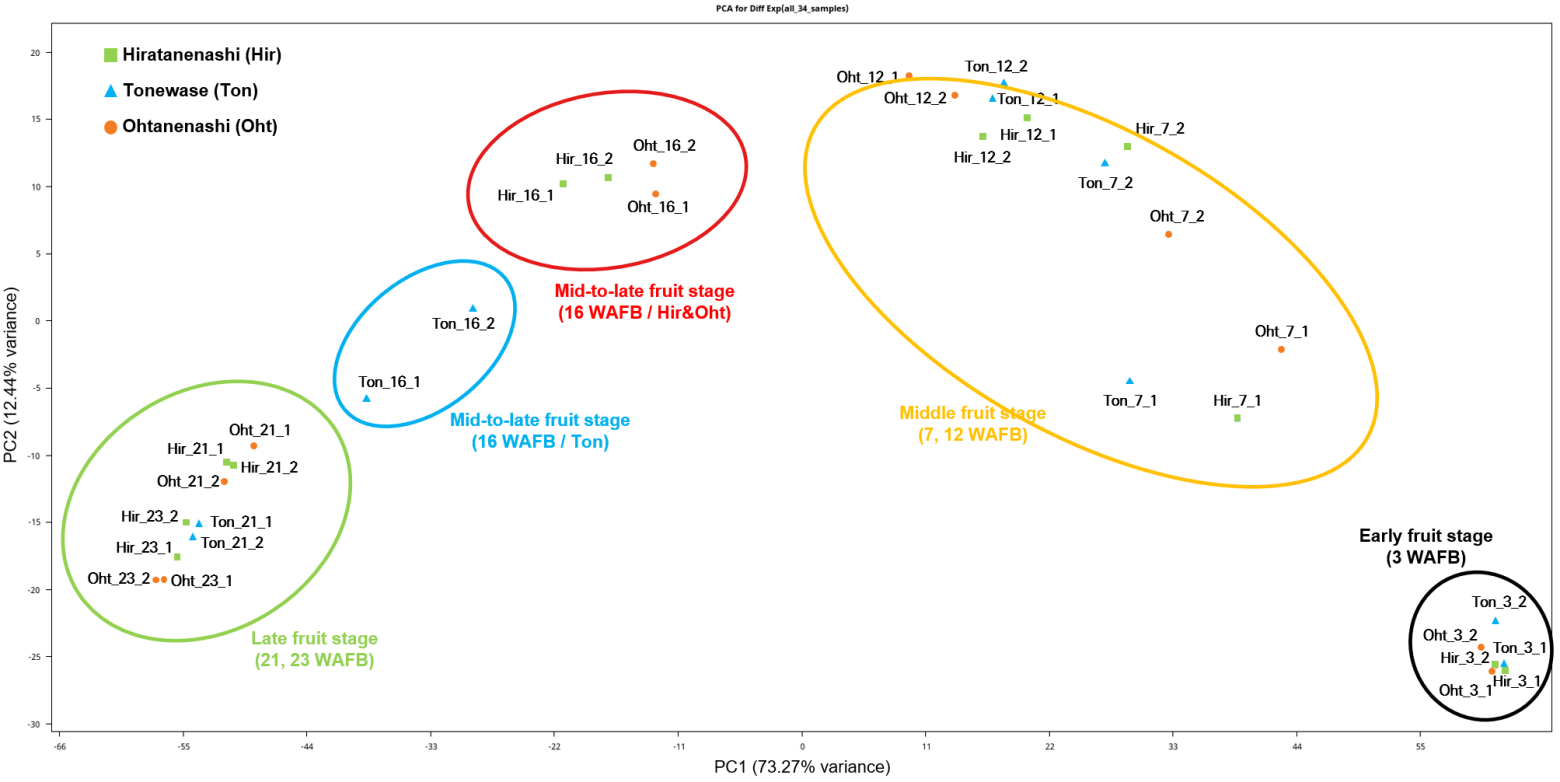
Transcriptome profiling of Hiratanenashi, Tonewase, and Ohtanenashi during fruit development. Principal component analysis (PCA) of RNA-seq samples from Hiratanenashi, Tonewase, and Ohtanenashi.

Principal component analysis (PCA) of the 34 samples, based on FPKM values, revealed a distinct inverted U-shaped distribution (**Figure 4**), indicating a clear differentiation pattern in gene expression profiles linked to various stages of fruit development. Notably, the early-stage (3 WAFB) and intermediate-stage (7 and 12 WAFB) samples were positioned on the right side of the distribution. Interestingly, during the mid-to-late fruit stage (16 WAFB), Hiratanenashi and Ohtanenashi samples clustered closely together, whereas Tonewase samples at 16 WAFB, which are known for early maturation, were located near to the late fruit stage (21 and 23 WAFB) samples.

Correlation analysis demonstrated strong correlations among replicates, with correlations ranging from 0.92 to 1 (**Supplementary Figure S2**). Samples collected at the same time points exhibited robust correlations. However, a significant observation emerged when analyzing Tonewase at 16 WAFB. This sample displayed a distinct correlation pattern, differing from Hiratanenashi and Ohtanenashi at the same stage, consistent with the findings of the PCA (**Figure 4**).

### 3.4 Co-expression gene network modules associated with fruit development

We conducted a comparative analysis of RNA-seq samples from adjacent developmental stages (3–7 WAFB, 7–12 WAFB, 12–16 WAFB, 16–21 WAFB, and 21–23 WAFB) in Hiratanenashi, resulting in the identification of 6,854, 1,808, 2,820, 6,876, and 3,010 DEGs, respectively (**Supplementary Figure S3**). For Ohtanenashi, the same stages yielded 5,801, 2,922, 2,937, 6,915, and 3,516 DEGs. In Tonewase, stages 3–7 WAFB, 7–12 WAFB, 12–16 WAFB, and 16–21 WAFB revealed 7,194, 852, 4,973, and 5,768 DEGs, respectively, with no data for the 21–23 WAFB stage. Additionally, DEG analysis was extended to compare Hiratanenashi and the mutants Ohtanenashi and Tonewase, revealing the following numbers of DEGs between Hiratanenashi and Ohtanenashi: 124 (3 WAFB), 270 (7 WAFB), 365 (12 WAFB), 247 (16 WAFB), 282 (21 WAFB), and 1,183 (23 WAFB). Similarly, there were 98 (3 WAFB), 531 (7 WAFB), 192 (12 WAFB), 1,937 (16 WAFB), 1,420 (21 WAFB), and 1,415 (Hir. 23 vs. Ton. 21 WAFB) between Hiratanenashi and Tonewase. In total, we identified 15,814 unique DEGs, which served as the basis for further analysis.

**Figure 5:**
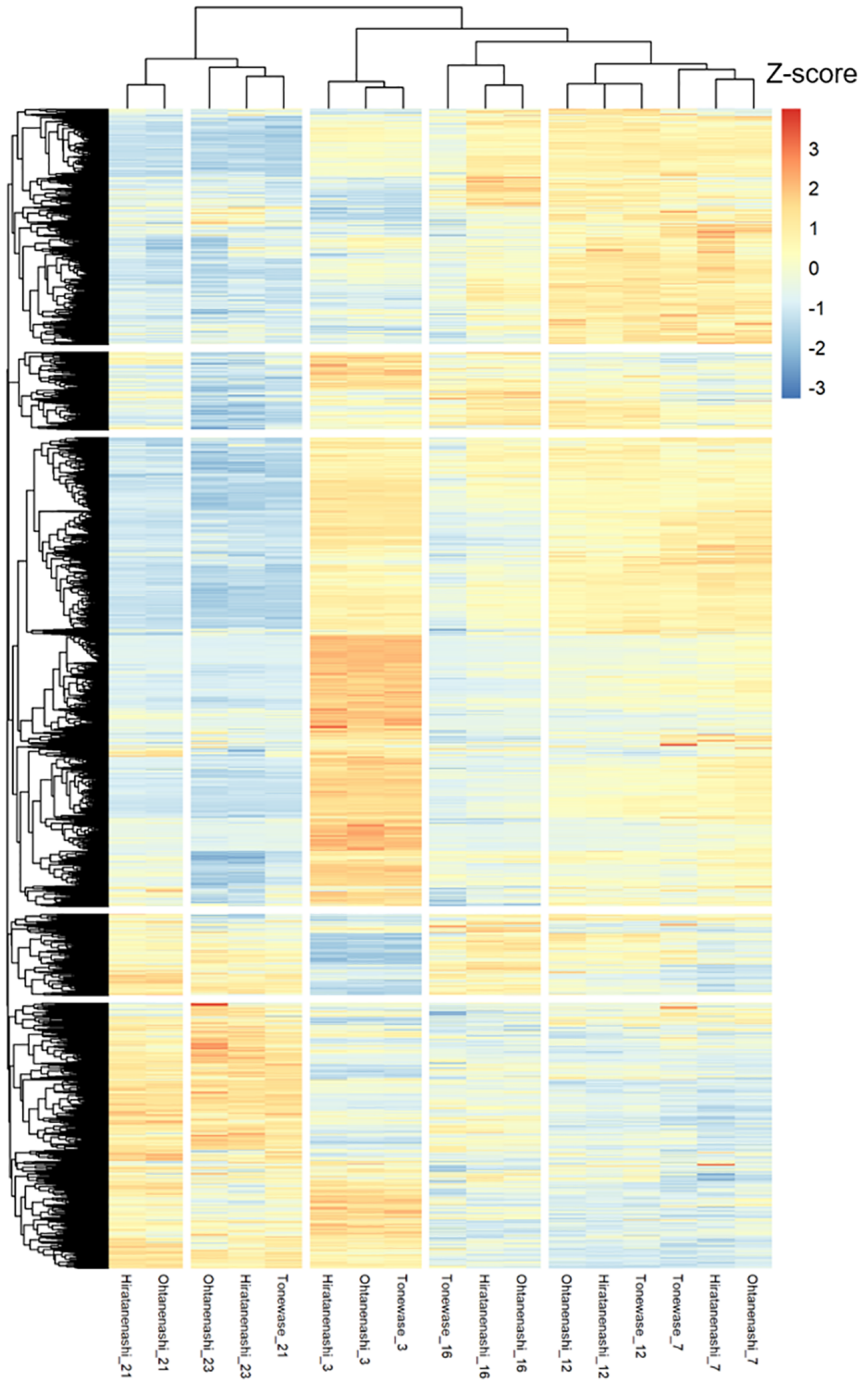
Hierarchical clustering and heatmap analysis of 15,814 differentially expressed genes (DEGs). The Z-score of Log_2_(FPKM + 1) was used for analysis.

**Figure 6:**
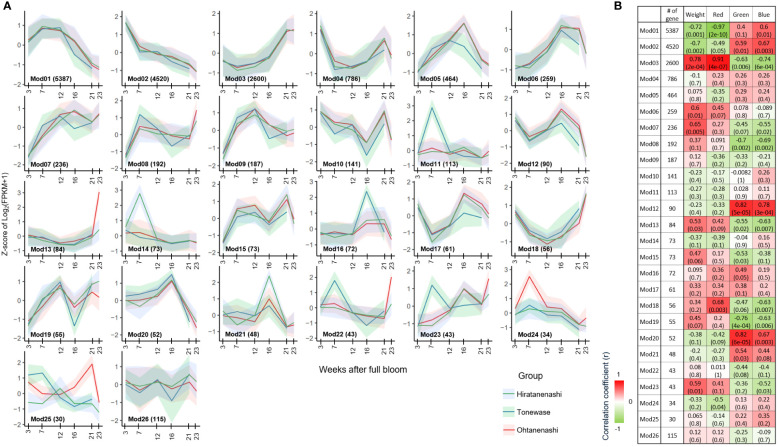
Weighted gene co-expression network analysis (WGCNA) of 15,814 differentially expressed genes (DEGs). **(A)** Gene expression profile of 26 identified modules from WGCNA. The x-axis represents weeks after full bloom, and the y-axis represents the Z-score of Log_2_(FPKM + 1) derived from RNA-seq data for each sampling point. The lines correspond to the cluster mean, and the shaded intervals correspond to the standard deviations. **(B)** Correlations between identified modules and traits. The correlation coefficient value displayed on top and corresponding p-value is presented in parentheses.

**Figure 7:**
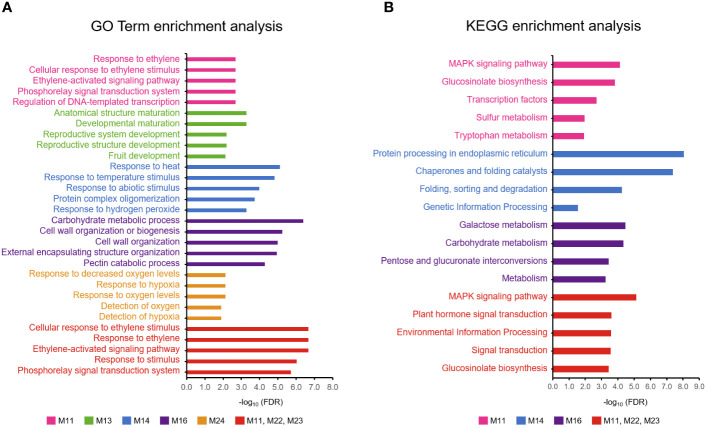
Enrichment analysis of modules showing cultivar-specific expression pattern. **(A)** Gene Ontology (GO) and **(B)** KEGG enrichment analysis. Top five enriched terms for each module were displayed.

Clustering analysis of these 15,814 DEGs indicated a clear division into two primary groups (**Figure 5**). The first group included DEGs from the early to mid-fruit developmental stages (3–16 WAFB), whereas the second group encompassed DEGs from the late fruit stage (21–23 WAFB). Within the first group, there were three subgroups corresponding to 3 WAFB, 7–12 WAFB, and 16 WAFB samples. In the second group, there were two subgroups, with the notable finding that Tonewase’s 21 WAFB samples clustered with the 23 WAFB samples of both Hiratanenashi and Ohtanenashi, suggesting a closer developmental timing. We performed a comprehensive WGCNA using 15,814 non-redundant DEGs to explore pathways associated with early fruit maturation in Tonewase and larger fruit size in Ohtanenashi, compared to those in their parent, Hiratanenashi (**Supplementary Figure S4**). This analysis led to the identification of 26 distinct WGCNA modules, designated Mod1–Mod26 (**Figure 6A**). To understand the relationships between these modules and the observed phenotypic traits, we conducted a module-trait correlation analysis (**Figure 6B**). Additionally, we carried out GO term and KEGG enrichment analyses to infer the potential functions of genes within specific modules (**Figure 7**).

Among the phenotypes examined, we identified modules significantly (p < 0.001) correlated with weight and red color, specifically Mod(M)1 (5,387 genes) and M3 (2,600 genes). M1 showed consistently decreased expression levels from 7 WAFB onward, with genes involved in processes such as metabolism and photosynthesis. This was supported by GO term enrichment analysis (GO:0008152, metabolic process, FDR P-value: 1.32E−64/GO:0009765, photosynthesis, light harvesting, FDR P-value: 5.98E−08) and KEGG pathway enrichment (00194 photosynthesis proteins, FDR P-value: 5.95E−06) (**Supplementary Files 1**, **2**). Conversely, genes within the M3 module exhibited increased expression levels from early to late fruit development and were associated with metabolism, as confirmed by the KEGG enrichment (A09100 Metabolism, FDR P-value: 9.28E−07) and GO term enrichment (GO:0008152, metabolic process, FDR P-value: 4.35E-35), affirming their relevance to the observed phenotypic traits.

The M2 module, containing 4,520 genes, did not show a significant association with any specific trait but exhibited declining expression levels from early fruit development to maturation. This module was primarily linked to cell division, as indicated by the significant enrichment of various GO and KEGG terms related to cell cycle processes (**Supplementary Files 1**, **2**). This finding suggests that the M1–M3 modules, which include 12,507 of the 15,814 DEGs (79%), are essential for fruit development, encompassing functions related to photosynthesis, cell cycle, and metabolism.

Modules M12 and M20 showed a significant correlation with green color component in the RGB color space, whereas M3 and M12 were correlated with the blue component. Although M12 did not display specific enrichment in KEGG or GO terms, M20 was significantly enriched in the GO term GO:0035672, associated with oligopeptide transmembrane transport (**Supplementary File 1**).

### 3.5 Cultivar-specific modules provide insight into fruit maturation and weight

Among the 26 modules identified, several exhibited unique expression patterns specific to the different cultivars during fruit development. Notably, M11, M16, M22, and M23 were uniquely expressed in Tonewase, whereas M13 and M24 showed distinct expression patterns in Ohtanenashi. Conversely, M14 had a unique expression pattern specific to the parent cultivar, Hiratanenashi. These modules were selected for further analysis based on the hypothesis that genes with unique expression patterns in particular cultivars are likely to contribute to the phenotypic differences observed (**Figure 6A**). We focused on genes with distinct expression profiles, those uniquely expressed or significantly upregulated in Tonewase, contributing to early maturation, and those associated with larger fruit size in Ohtanenashi, to uncover the genetic basis of these cultivar-specific traits. This approach sheds light on the molecular mechanisms driving significant phenotypic variations, emphasizing the role of cultivar-specific gene expression in these differences.

Among the modules specific to Tonewase, M11, M22, and M23 were upregulated at 7 WAFB compared to the other samples. Particularly, M11 stood out with significant enrichment in both GO and KEGG terms (**Figure 7**). The top three GO terms associated with M11 were “Response to ethylene,” “Cellular response to ethylene stimulus,” and “Ethylene-activated signaling pathway.” This module included various ethylene-related genes such as *ethylene receptor 2* (*DKAch13a03800.t1*), *ethylene overproduction protein 1* (*DKAch09a29729.t1*), *ethylene-responsive transcription factor 1B-like* (*DKAch07a36630.t1*, *DKAch15×12633.t1*, and *DKAch15y10353.t1*), *ethylene-responsive transcription factor 4-like* (*DKAch03a24844.t1*), *ethylene-responsive transcription factor 5-like* (*DKAch02a09633.t1*), and *ethylene-responsive transcription factor ABR1-like* (*DKAch15x13695.t1* and *DKAch15y11457.t1*). Another Tonewase-specific module, M16, showed upregulation at 16 WAFB and was enriched in genes related to carbohydrate synthesis and cell wall processes (**Figure 7A**).

For Ohtanenashi, modules M13 and M24 exhibited unique expression patterns. M13 was specifically upregulated at the Ohtanenashi maturation date (23 WAFB) (**Figure 6A**) and was enriched in numerous GO terms related to fruit development and maturation (**Figure 7A**). In contrast, M24 showed upregulation at 7 WAFB in Ohtanenashi, with enrichment analysis indicating various stress response-related GO terms (**Figure 7A**). Given the observed differences in fruit size from the early stages of development, the expression pattern in M24 suggests these genes likely influence fruit size. Genes within M24, such as *scarecrow-like protein 9*, *transcription factor WRKY19-like*, and *ethylene-responsive transcription factor ERF011-like*, are known for their roles in plant development, including the regulation of fruit size. We conducted a comparative chromosomal positions analysis of the 34 genes in M24 to investigate whether they were closely located. Among these, 24 genes were identified near chromosomes 1 (five genes), 3 (two genes), 4 (two genes), 9 (three genes), 10 (five genes), 11 (five genes), and 12 (two genes) (**Supplementary File 2**, **Supplementary Figure S5**). Module M14 exhibited expression patterns specific to Hiratanenashi, showing upregulation at 7 WAFB (**Figure 6A**). The GO term and KEGG enrichment analyses indicated that M14 contained genes related to stress responses and protein functions (**Figure 7**). Additionally, module M25 displayed unique expression patterns across all samples although it did not show any enriched KEGG or GO terms (**Figure 6A**).

**Figure 8:**
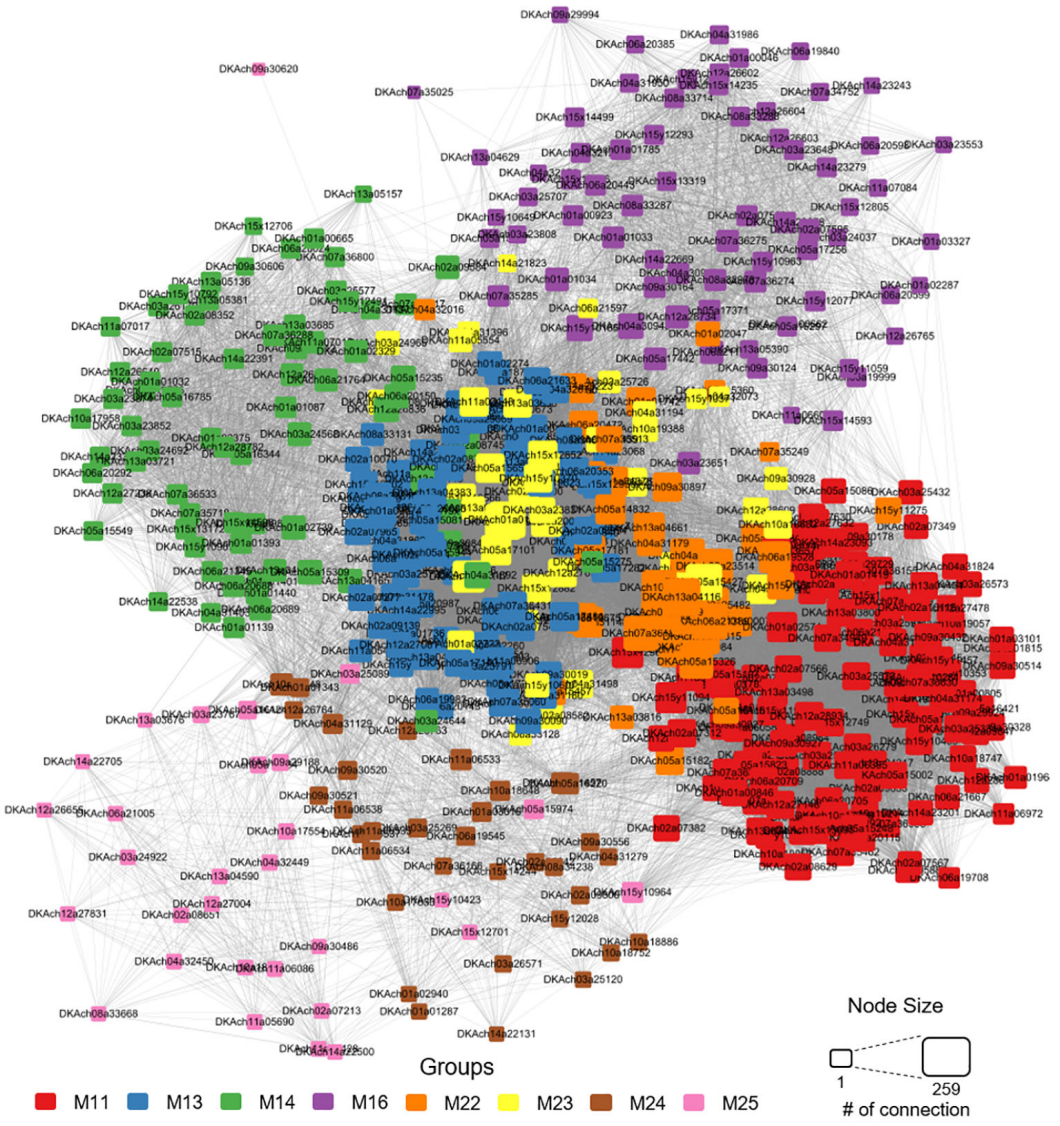
Network analysis based on co-expression analysis within the cultivar-specific modules (M11, M13, M14, M16, M22, M23, M24, and M25). Genes are color-coded according to their respective modules, and node size reflects the number of edges connecting them to other nodes.

We focused on gene networks within modules M11, M13, M14, M16, M22, M23, M24, and M25, which displayed distinctive expression patterns among the cultivars and were likely to influence fruit size or maturation dates. As expected, genes within the same module were closely connected (**Figure 8**). Despite being part of different modules, M11, M22, and M23, each upregulated at 7 WAFB in Tonewase, were positioned close to each other, reinforcing their interconnected roles. GO terms and KEGG enrichment analyses on all genes in these three modules revealed a higher enrichment of multiple ethylene-related GO terms compared to M11 alone, as indicated by lower FDR P-values (**Figure 7**).

**Figure 9:**
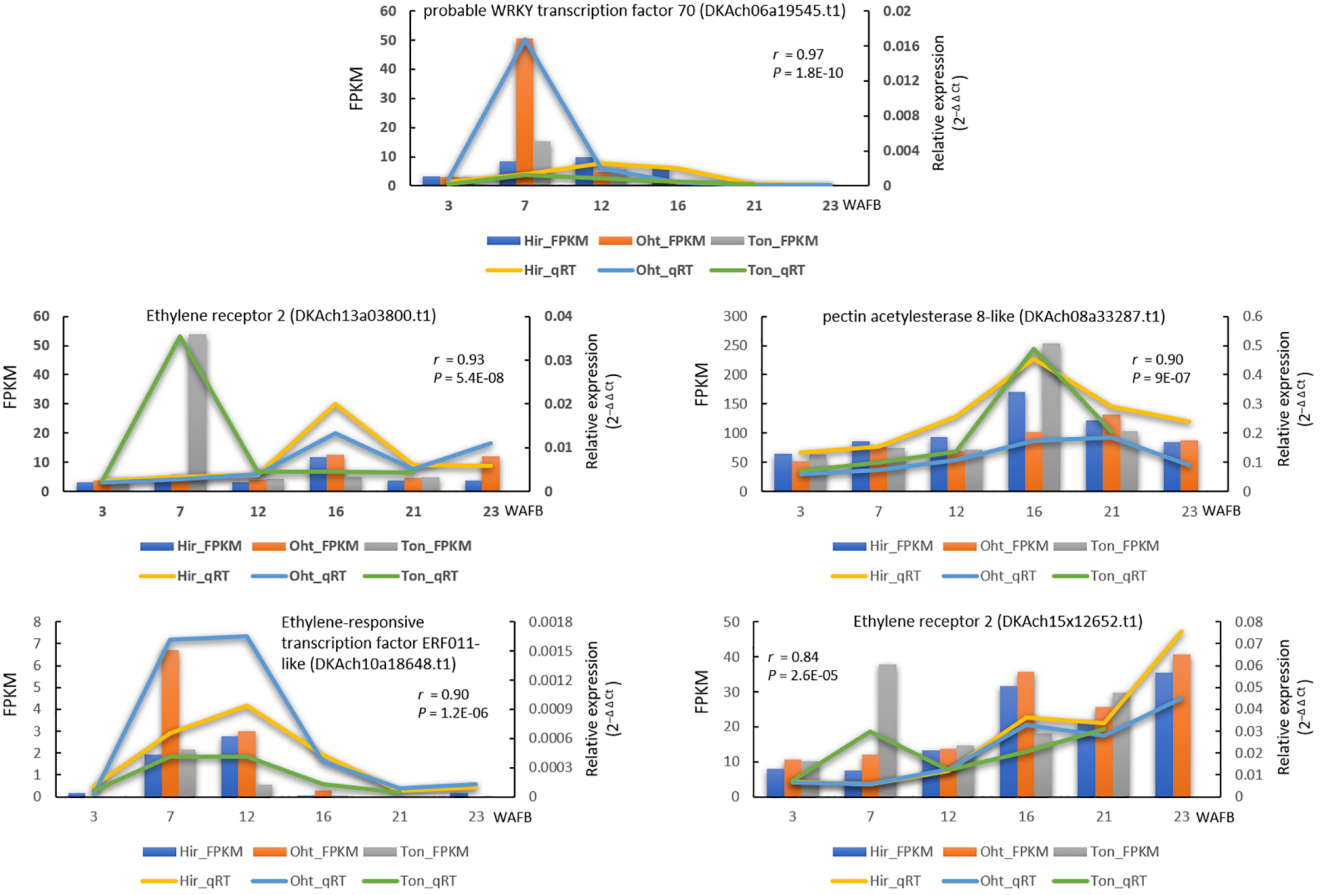
Gene expression comparison of five genes between RNA-seq and qRT-PCR during fruit development. The lines indicate qRT-PCR relative expression data on the right y-axis, whereas the columns represent RNA-seq FPKM data on the left y-axis, with the time in weeks after full bloom (WAFB) along the x-axis. For qRT-PCR, relative expression levels were normalized against the control gene (GADPH) using the 2^−ΔΔCt^ method. A Pearson correlation was performed between the mean qRT-PCR values (from two biological replicates, each with three technical repeats) and RNA-seq data (from two biological replicates), with the resulting correlation coefficient and p-value noted.

To validate the accuracy of our RNA-seq results, we performed qRT-PCR analyses on five selected DEGs that exhibited cultivar-specific expression patterns. The comparison between the FPKM values from RNA-seq and the relative expression (2^–Δ Δ Ct)^values from qRT-PCR showed Pearson correlation values ranging from 0.84 to 0.97 (**Figure 9**). This demonstrates a high reliability of our RNA-seq data.

## 4 Discussion

### 4.1 Establishing the relationship between bud sports and their parent using GBS

Fruit size and maturation are complex traits influenced by the coordinated activity of multiple genes during fruit development, making it challenging to unravel their molecular mechanisms. Unlike model plants such as Arabidopsis, where transfer DNA (T-DNA) insertion lines are commonly used, studying these mechanisms in fruit tree is hindered by difficulties in transformation and low regeneration efficiency (Habu et al., 2016). Consequently, researchers have shifted their focus to exploring naturally occurring bud sport mutations. Bud sports, almost genetically identical to their original parent except for the mutated gene(s) or region, offer valuable research materials (Ban et al., 2022). However, it is essential to confirm the relationship between the bud sport and its parent to ensure the accuracy of the study materials.

Previous attempts to analyze the genetic diversity of persimmon accessions, including Hiratanenashi, using SSR markers, involved 495 accessions (Guan et al., 2020) and 76 accessions (Wang et al., 2020b). Unfortunately, these studies did not include the bud sports of Hiratanenashi, Tonewase, and Ohtanenashi. Conventional bud sport studies typically validate the bud sport alongside a few well-known varieties for comparison. However, we successfully established the relationship between the bud sport and its parent by utilizing over 50,000 SNPs obtained through GBS across 192 samples. Our results revealed that Hiratanenashi and its bud sport samples, including Tonewase and Ohtanenashi, formed a single clade, with A24 (Uljinmulgam) also grouped together. The ploidy test confirmed that the A24 cultivar is a nonaploid (9x) like Hiratanenashi, suggesting that A24 could be another bud sport of Hiratanenashi; however, further validation is required.

### 4.2 Identification of metabolic pathways involved in fruit maturation

Bud sports that exhibit earlier or delayed maturation compared to their parent plants have been observed in various species. Numerous studies consistently emphasize the pivotal role of the hormone ethylene in the maturation process, particularly in climacteric fruit species (Klee and Giovannoni, 2011; Liu et al., 2015). Additionally, research suggests that sensitivity to the ethylene response varies among different genetic backgrounds (Paul et al., 2012). In our study, the early maturing Tonewase mutant displayed significant differences in ethylene-related pathway genes compared to the other two cultivars (**Figures 6A**, **7**), indicating that ethylene likely plays a crucial role in the early maturation of Tonewase.

We have identified the M11 module as a key player in the early maturation of Tonewase, characterized by distinct upregulation at 7 WAFB and comprising ethylene-related genes (**Figure 6A**). Further insights from the network analysis revealed a tight interconnection among modules M11, M22, and M23, which all displayed specific upregulation in Tonewase at 7 WAFB (**Figure 8**). The close interconnection between these three modules, coupled with the significant enrichment of ethylene-related GO and plant hormone KEGG terms (**Figure 7**), underscores the collaborative nature of these modules in orchestrating the early maturation process in Tonewase. This is consistent with findings in other species. For instance, the bud sport “Beni Shogun” apples, which mature earlier than the parent “Fuji,” exhibit increased expression of ethylene synthesis and signal transduction genes (Dong et al., 2011). Similarly, research on clementine mandarins has shown that differences in maturation date are attributed to altered expression of ethylene-related genes between the bud sport and its parent (Distefano et al., 2009; Alos et al., 2014).

Research comparing bud sports with their parent plants also suggests that the regulation of cell wall-related genes influences maturation. For example, a late-maturing mutant of sweet orange exhibited differential expression of various hormone pathways and cell wall-related genes compared to the original cultivar (Wu et al., 2014). This is further supported by observations in apples (Wang et al., 2009), where differential gene expression related to ethylene biosynthesis and signaling, along with a cell wall degradation enzyme, was highlighted as a key factor leading to distinct maturation dates between the mutant and its parent.

Another noteworthy module, M16, was recognized as a potential contributor to the observed differences in fruit maturation (**Figure 6A**). This module’s distinctive expression pattern, particularly the upregulation observed in Tonewase at 16 WAFB, and the significant enrichment of several cell wall-related pathways (**Figure 7**) emphasize its potential role in fruit maturation.

In summary, our exploration of the molecular landscape of the accelerated maturation in Tonewase revealed key modules such as M11, M22, M23 (ethylene-related), and M16 (cell wall). These findings enhance our understanding of the specific genetic factors influencing maturation and lay the groundwork for future investigations into the complex network of processes shaping fruit development.

### 4.3 Identification of metabolism pathways involved in fruit size

Bud sports with larger or smaller fruit sizes than their parent plants are frequently identified due to their distinct characteristics. Notably, the fruit of the bud cultivar Ohtanenashi was more than twice the size of its parent cultivar Hiratanenashi at harvest (**Figure 2B**). Fruit development involves both cell division and expansion, which collectively determine the final fruit size. A study examining changes in cell size during fruit development in Ohtanenashi and Hiratanenashi revealed that Ohtanenashi consistently displayed larger cell numbers and sizes compared to Hiratanenashi (Hamada et al., 2015). Previous studies on bud sports highlighted the role of cell cycle-related gene expression in determining variations in fruit size. For instance, the “Grand Gala” (GG) apple mutant exhibited increased fruit size associated with enhanced cell size and ploidy, with significant changes in *MdCDKA1* and *MdCYCA2* gene expressions during early fruit development (Malladi and Hirst, 2010). Conversely, the somatic mutant “Hasshu” of Hiratanenashi, which exhibits a small fruit size phenotype, showed a decrease in ploidy level (Yakushiji et al., 2016). Although changes in ploidy level leading to alterations in fruit size have been reported, Ohtanenashi, maintained a consistent ploidy level with the original cultivar (**Figure 2A**). In the case of the Giant La France pear, a bud sport of the European pear “La France” producing enlarged fruit, 61 core cell cycle genes were identified, although their specific roles in the increased fruit size remains unclear (Nashima et al., 2014). Additionally, in another giant pear bud sport, elevated expression of an actin-related protein, which regulates cell proliferation in Arabidopsis, was observed compared to the original cultivar (Deal et al., 2005; Zhang et al., 2015).

Considering the observed differences in fruit size between Hiratanenashi and Ohtanenashi in our experiment and variations in cell size and number from previous studies, genes displaying distinct expression patterns in Ohtanenashi during early fruit development, identified in the M24 module (**Figure 6A**), emerged as key contributors to the observed differences in fruit size (**Figure 2**). Among the 34 genes in the M24 module, five were located on chromosome 1 (**Supplementary File 2**, **Supplementary Figure S4**). Notably, three genes—*DKAch01a01287.t1* (unannotated), *DKAch01a01343.t1* (*scarecrow-like protein 9*), and *DKAch01a01457.t1* (*E3 ubiquitin-protein ligase AIRP2*)—were closely positioned. The other two genes—*DKAch01a02940.t1* (*endoglucanase 25-like*) and *DKAch01a03016.t1* (*transcription factor WRKY19-like*)—were also located near each other. This spatial clustering on the chromosome suggests potential functional interrelations and shared regulatory mechanisms. Gene clustering, especially for those involved in related biological processes or signaling pathways, often indicates co-expression facilitated by physical proximity (Makino and McLysaght, 2012). Whereas DKAch01a01287.t1 is unannotated, the remaining genes are associated with critical functions related to the cell cycle and growth.

The *scarecrow*-like protein 9 gene is crucial for establishing the radial organization of the *Arabidopsis* root by regulating asymmetric cell division, which belongs to the *SCR* family of putative transcription factors (Di Laurenzio et al., 1996; Wysocka-Diller et al., 2000; Heidstra et al., 2004). It is expressed in cortex/endodermal initial cells and the endodermal cell lineage and plays a vital role in the transcriptional regulation of tissue-specific expression. Additionally, the E3 ubiquitin-protein ligase *AIRP2* is essential for lateral root development and regulates plant phytohormone biosynthesis, transport, signaling pathways, and cell cycle progression (Koiwai et al., 2007; Prasad et al., 2010; Yang et al., 2021). In addition, three *WRKY* transcription factor genes (*DKAch01a03016.t1*, *DKAch02a09800.t1*, and *DKAch06a19545.t1*) have a broad impact on fruit development (Bakshi and Oelmuller, 2014), and one ethylene-responsive transcription factor gene (DKAch10a18648.t1) were identified. Although further research is necessary to fully elucidate the impact of these genes on persimmon fruit development, the genes identified within the M24 module, despite not being enriched in GO terms or KEGG pathways, are considered potential candidates that may influence the fruit size of Ohtanenashi.

## 5 Conclusion

**Figure 10:**
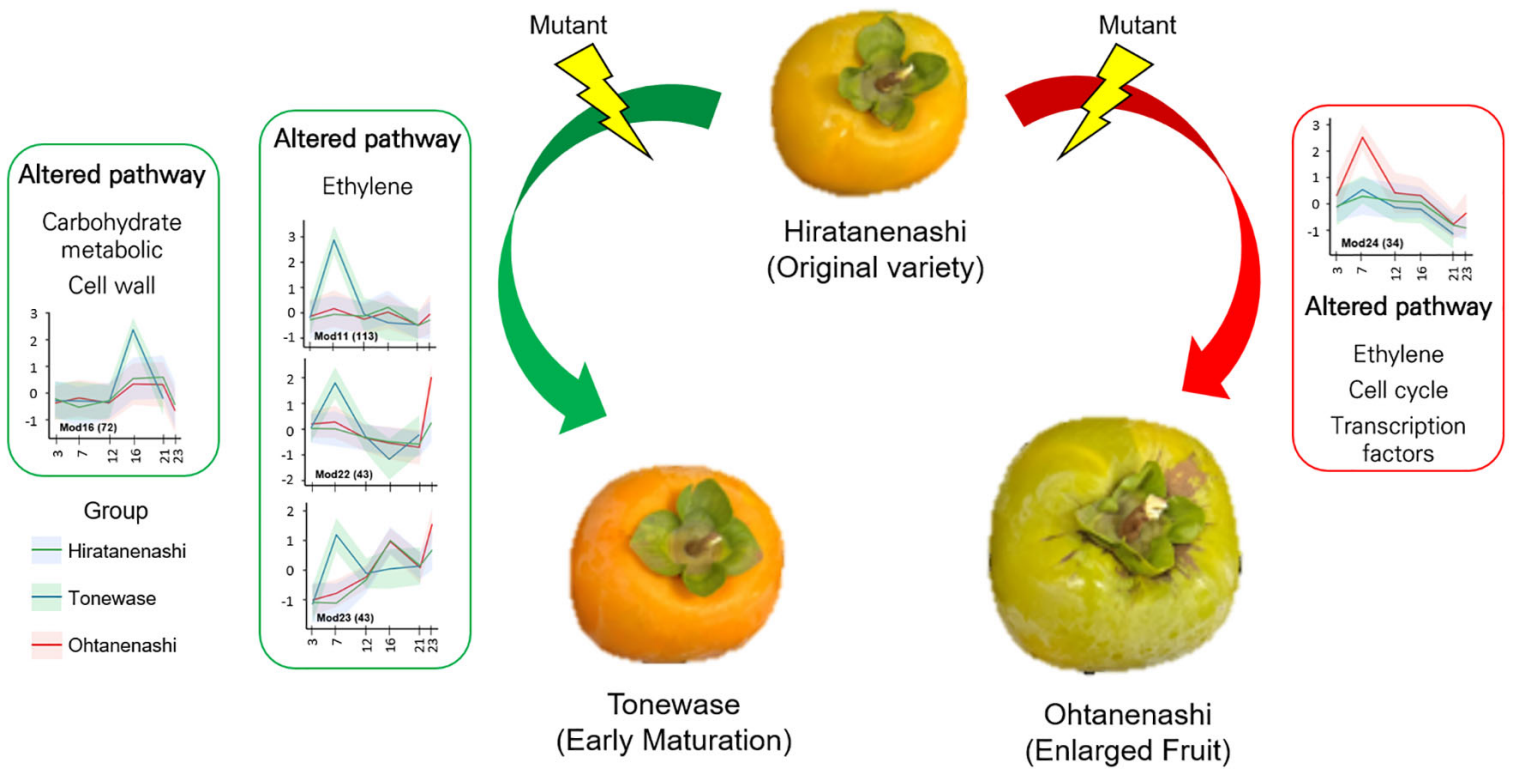
Proposed molecular mechanisms underlying the characteristics of bud sports Tonewase (early maturation) and Ohtanenashi (enlarged fruit).

In summary, we conducted a comparative transcriptome analysis to explore the differences in gene expression during fruit development between bud sport samples and their original cultivar, Hiratanenashi. This analysis highlighted potential pathways and genes that may contribute to the observed variations in fruit size and maturation. The accelerated maturation in Tonewase was associated with the altered expression of genes in the ethylene and cell wall pathways, suggesting their involvement in early fruit ripening (**Figure 10**). Specifically, the upregulation of ethylene-related genes in the M11 module appears to play a key role in this process. For Ohtanenashi, the larger fruit size was linked to genes in the M24 module, particularly those involved in the cell cycle, ethylene signaling, and transcription factors (**Figure 10**). The spatial clustering of these genes on chromosome 1 hints at a coordinated regulatory mechanism that may enhance fruit growth. Although we did not perform direct validation of these genes, our findings provide valuable insights into which gene functions might contribute to the phenotypic changes observed in these bud sports compared to their original cultivar. Future studies will be necessary to characterize these candidate genes and verify their functions through additional gene characterization experiments. This study lays the groundwork for understanding and potentially manipulating these traits to improve fruit quality in persimmons.

## Data availability statement

The datasets generated for this study can be found in the NCBI-SRA repository, accession number PRJNA1056501.

## Author contributions

SB: Writing – review & editing, Writing – original draft, Visualization, Investigation, Funding acquisition, Formal analysis, Data curation, Conceptualization. H-yS: Writing – review & editing, Visualization, Investigation, Data curation. SL: Writing – review & editing, Validation, Investigation. S-HK: Writing – review & editing, Validation, Investigation. SO: Writing – review & editing, Validation, Formal analysis. JJ: Writing – review & editing, Supervision, Resources, Project administration, Funding acquisition.
