# Supplementary figures and images for: Comparative transcriptome analysis of persimmon somatic mutants (Diospyros kaki) identifies regulatory networks for fruit maturation and size
Source: Front Plant Sci. 2024 Aug 2;15:1448851. doi: 10.3389/fpls.2024.1448851 (PMC11327018; doi:10.3389/fpls.2024.1448851)

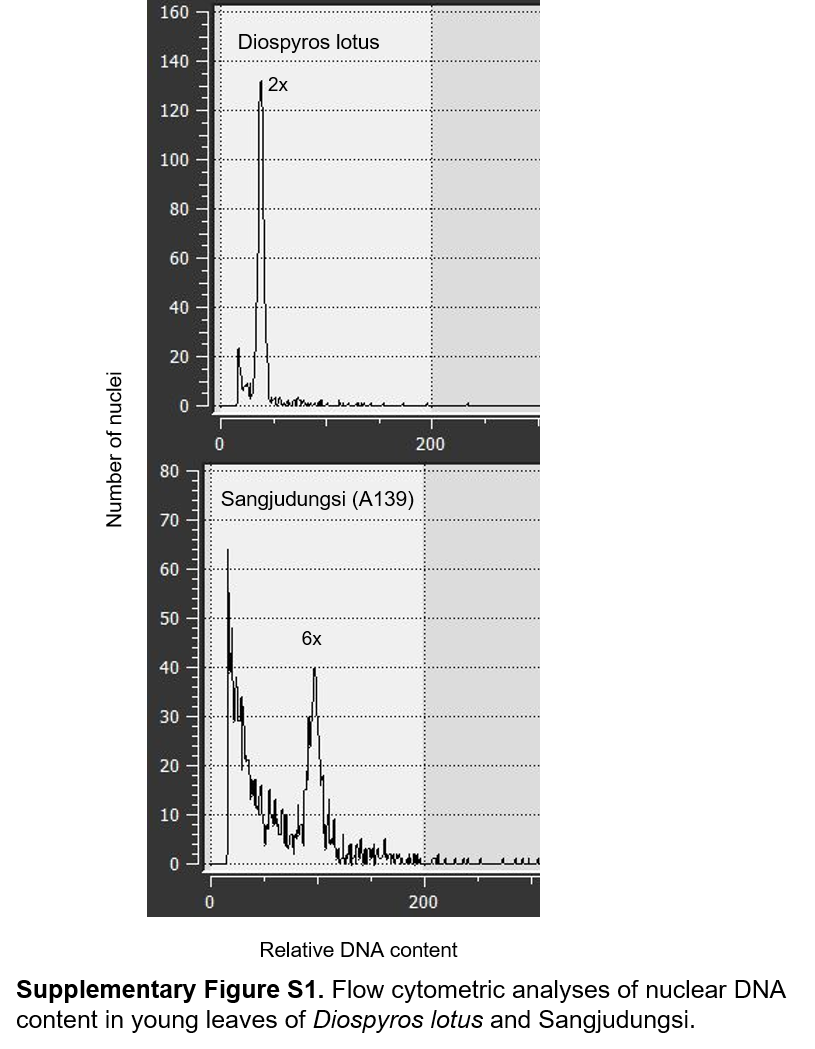

Supplement: Supplementary file 1 [file Image_1.tif]

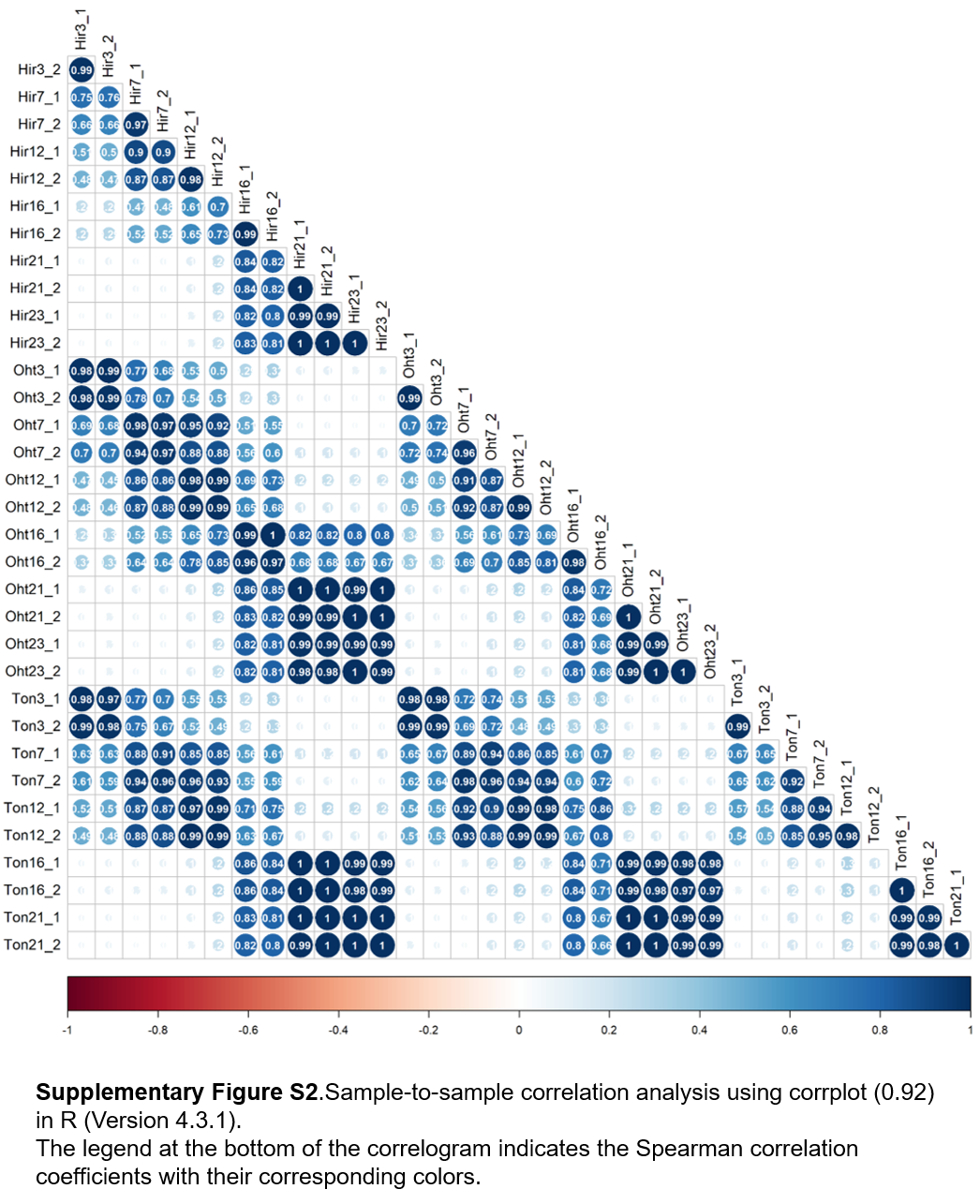

Supplement: Supplementary file 2 [file Image_2.tif]

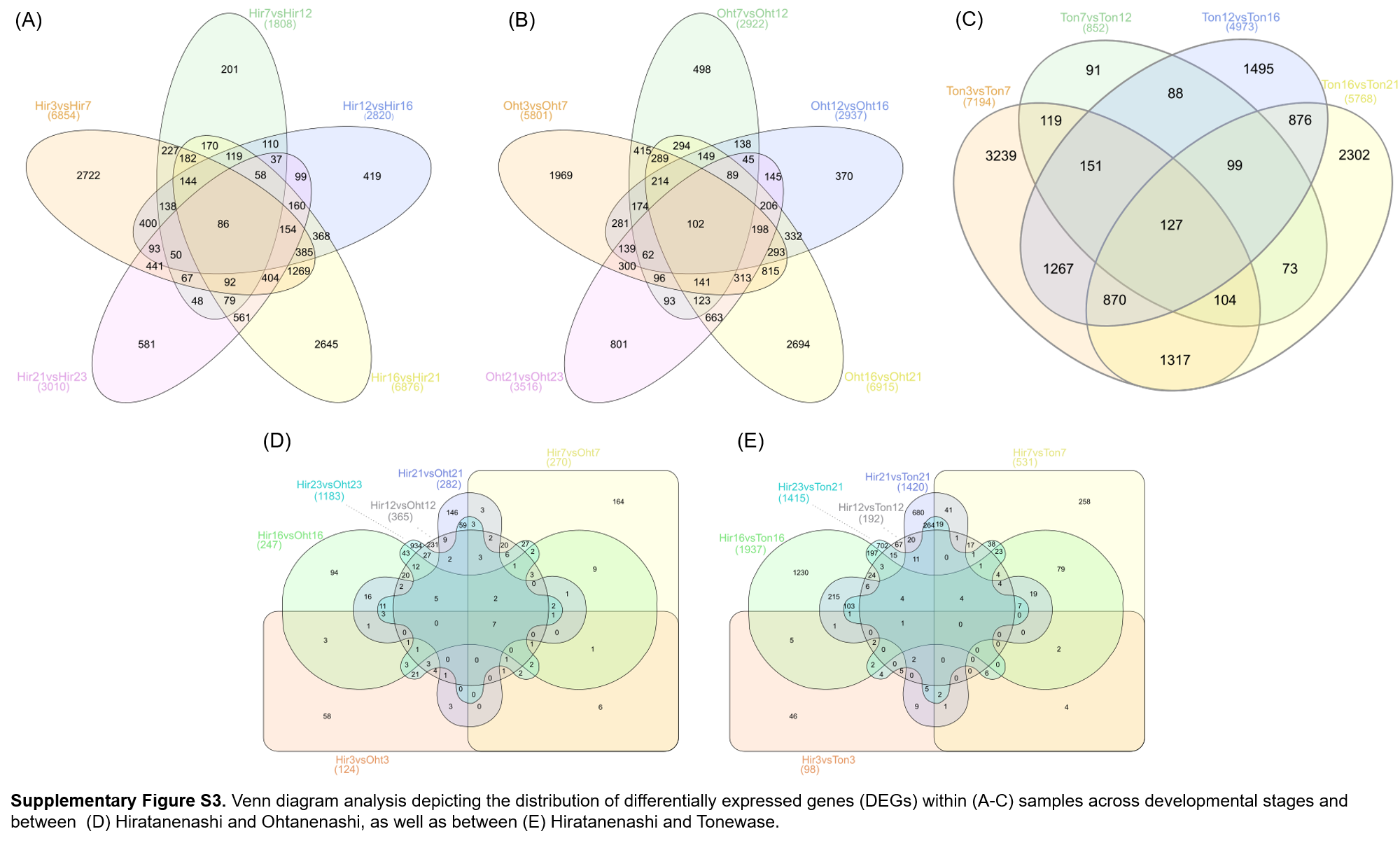

Supplement: Supplementary file 3 [file Image_3.tif]

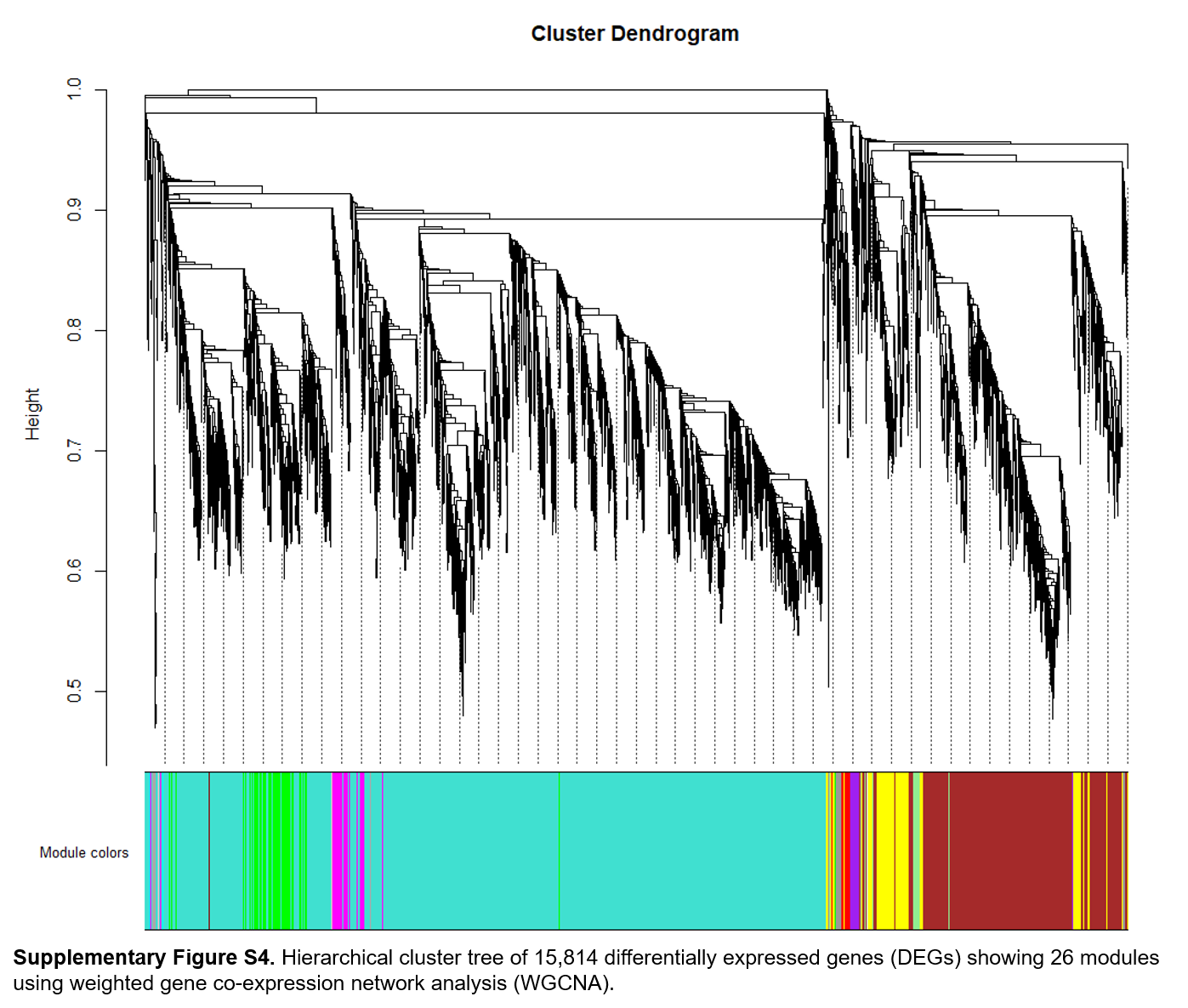

Supplement: Supplementary file 4 [file Image_4.tif]

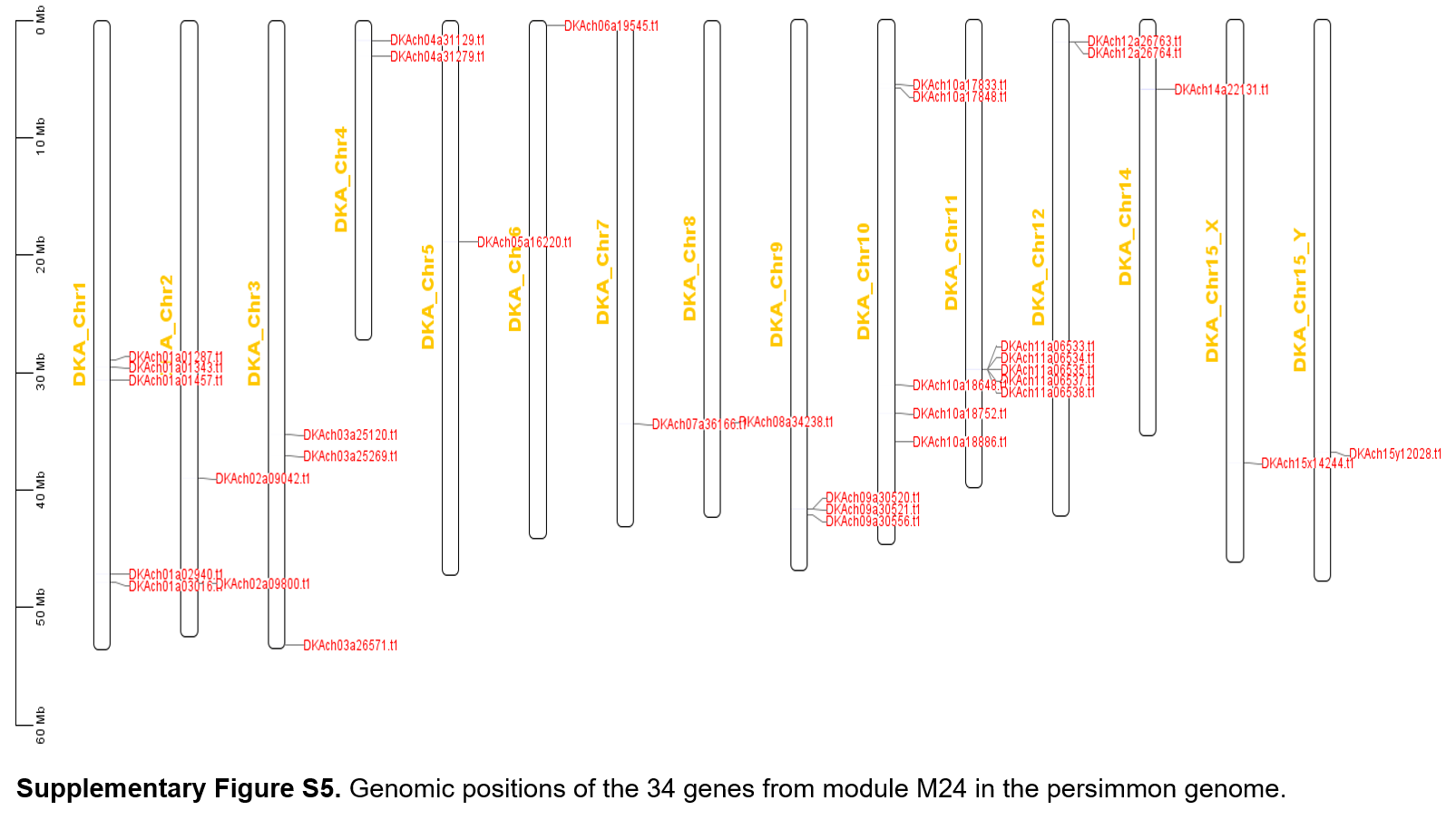

Supplement: Supplementary file 5 [file Image_5.tif]
